# An overview of functional genomic tools in deciphering insecticide resistance

**DOI:** 10.1016/j.cois.2018.04.004

**Published:** 2018-06

**Authors:** Rafael A Homem, Thomas G Emyr Davies

**Affiliations:** Department of Biointeractions and Crop Protection, Rothamsted Research, Harpenden AL5 2JQ, UK

## Abstract

In this short review, we highlight three functional genomic technologies that have recently been contributing to the understanding of the molecular mechanisms underpinning insecticide resistance: the GAL4/UAS system, a molecular tool used to express genes of interest in a spatiotemporal controlled manner; the RNAi system, which is used to knock-down gene expression; and the most recently developed gene editing tool, CRISPR/Cas9, which can be used to knock-out and knock-in sequences of interest.

## Highlights

• Functional genomics have been key to deciphering mechanisms of insecticide resistance.
• The model organism *D. melanogaster* has played a central role in these studies.
• UAS/GAL4 and RNAi are the two main technologies that have been extensively deployed.
• CRISPR/Cas9 editing now facilitates functional validation of resistance mechanisms.

**Current Opinion in Insect Science** 2018, **27**:103–110

This review comes from a themed issue on **Pests and resistance**

Edited by **Chris Bass** and **Christopher Jones**

For a complete overview see the Issue and the Editorial

Available online 13th April 2018

**https://doi.org/10.1016/j.cois.2018.04.004**

2214-5745/© 2018 The Authors. Published by Elsevier Inc. This is an open access article under the CC BY license (http://creativecommons.org/licenses/by/4.0/).

## Introduction

Functional genomic technologies make use of the data produced by genomic and transcriptomic projects to try to elucidate the role played by genes of interest in *in vivo* systems. This can be done by systematically knocking-down, knocking-out or over-expressing specific targets. Not surprisingly, due to the vast array of functional genomic tools available, *Drosophila melanogaster* has been at the forefront of these studies. However, advances in germline transformation technologies in non-model insects and the development of technologies that do not require germline transformation have recently expanded the applicableness of functional genomics. Here we briefly review these technologies and how they have been applied to the study of the mechanisms of insecticide resistance in insect pests and disease vectors.

## The GAL4/UAS system

**Figure 1:**
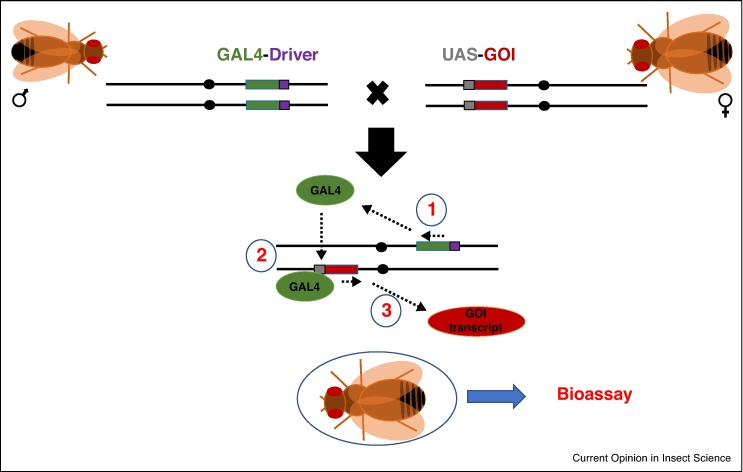
The GAL4/UAS binary targeted gene expression system. The system consists of a transgenic strain in which coding sequence for the yeast transcription factor, GAL4, is under the control of a promoter or enhancer of interest, Driver, and a second transgenic strain in which the GAL4 target, Upstream Activating Sequence (UAS), controls transcription of a gene of interest (GOI). GOI is only transcribed in the F1 progeny from these crosses in which one copy of each construct is present. In the F1 progeny, GAL4 is produced (1), binds to the UAS (2) and activates the expression of GOI (3). F1 flies are used in bioassays.

Nearly 20 year ago Fischer *et al.* demonstrated that it was possible to make use of the yeast transcription factor GAL4 in the fruit fly *D. melanogaster* to activate the expression of a reporter gene inserted next to an upstream activation sequence (UAS) [1]. This work paved the way for the development of one of the most powerful functional genomics technologies, the GAL4/UAS system [2]. In their landmark work, Brand & Perrimon developed a binary system that allows spatiotemporal control of targeted gene expression in *D. melanogaster*. The system can be used to express any gene of interest (GOI), including lethal ones, as GAL4-drivers and UAS-GOI constructs are usually integrated in separate transgenic strains (Figure 1). The authors then took another major step forward by generating a library of driver strains expressing GAL4 under the control of random enhancer sequences found in the genome of *D. melanogaster*. By further screening this library with the help of a *UAS-LacZ* reporter line, they could identify the embryonic expression pattern driven by some of these enhancers. Since then, a vast number of ‘trapped’ enhancer GAL4 strains have been generated and are now available for the scientific community (for a comprehensive review of the GAL4/UAS system see [3, 4]).

In pioneering work investigating the resistance of wild populations of *D. melanogaster* to dichloro-diphenyl-trichloroethane (DDT), Darbon *et al.* used the GAL4/UAS system to demonstrate that a single cytochrome P450 gene, *CYP6g1*, which was differentially expressed in a DDT resistant population, was responsible for conferring resistance to that insecticide [5]. By overexpressing *UAS-CYP6g1* under the control of a heat-shock inducible GAL4 driver (*Hsp-GAL4*) and showing that these flies became more resistant to DDT than control flies, the authors provided a clear correlation between *CYP6g1* expression and resistance to DDT. In a subsequent study, the overexpression of *UAS-CYP6g1* under the control of a tubulin GAL4 driver (*TubP-GAL4*) was used to demonstrate that, in addition to DDT, this P450 conferred cross-resistance to the organophosphorus (OP) compound malathion and to the neonicotinoid insecticides, acetamiprid, imidacloprid and nitenpyram [6]. Later it became clear that the insecticide resistance phenotype associated with *CYP6g1* was mainly due to the insertion of the long terminal repeat (LTR) of an *Accord* retrotransposon upstream of the gene, resulting in an increased *CYP6g1* expression in major detoxification tissues. To confirm the role played by the *Accord* LTR in DDT resistance, flies expressing *UAS-CYP6g1* under the control of an *Accord LTR-GAL4* driver (*6g1*HR-GAL4-*6c*) were shown to become more resistant to insecticides compared to control flies [7].

There are now numerous further examples of the use of the GAL4/UAS system in *D. melanogaster* to assess the contribution of individual detoxification enzymes to resistance in pest insects. GAL4-driven expression of *CYP12a4* to the midgut and Malpighian tubules of fruit flies resulted in resistance to the insect growth regulator lufenuron [8]. The GAL4 system has additionally been used to functionally validate three distinct detoxification enzymes from three biologically different pests: a carboxylesterase gene (*aE7*) conferring resistance to OPs in the Australian sheep blowfly, *Lucilia cuprina*; a glutathione S-transferase gene (*GstE2*) from the malarial mosquito, *Anopheles gambiae*, conferring resistance to DDT; and a cytochrome P450 gene (*CYP6cm1*) from the silverleaf whitefly, *Bemisia tabaci*, responsible for resistance to imidacloprid [9]. It was further employed to confirm the role of two alleles of the P450 genes *CYP6P9a* and *CYP6P9b* in driving resistance to pyrethroids in field populations of the malaria vector *Anopheles funestus* [10], and to demonstrate that overexpression of the glutathione S-transferase gene, *GSTe2*, caused resistance to DDT [11]. Moreover, the expression of the P450 gene *CYP6ER1* in transgenic flies under the control of the GAL4/UAS system demonstrated that it is responsible for strong resistance to the neonicotinoid insecticide imidacloprid in the brown planthopper *Nilaparvata lugens*, a major rice pest [12^•^]. A follow-up study showed that *CYP6ER1* is duplicated in resistant brown planthopper strains, with individuals carrying paralogs with and without the gain-of-function mutations responsible for conferring imidacloprid resistance [13].

Examples of the use of the GAL4/UAS system in insects other than *D. melanogaster* are rarer and the reasons for that can be related to three main constraints of non-model insects — technical difficulties of keeping large numbers of mutant stocks, unavailability of transformation technologies and husbandry protocols, and scarceness of genomic data. Despite these difficulties the technology has been developed in a few other insects. As early as 2003, Imamura *et al*. reported the establishment of a GAL4/UAS binary expression system in the silkworm *Bombyx mori* [14]. This moth-based transformation system has been further refined by studies evaluating the transcription-activation efficiency of different GAL4 variants [15] and, more recently, optimising transcriptional and translational enhancers to improve *in vivo* heterologous protein expression [16]. GAL4–UAS has also been developed in the red flour beetle, *Tribolium castaneum*, using established GAL4 variants [17], and in several mosquito species (*Anopheles gambiae*, *Anopheles stephensi, Aedes aegypti*), which are important insect vectors of human disease, to regulate the expression of integrated transgenes [18, 19, 20, 21]. GAL4–UAS was most recently employed to investigate the regulation of a gut-specific carboxypeptidase gene expression in *Aedes aegypti* [21], but will also have utility in the future to investigate insecticide resistance mechanisms.

## The success and challenges of RNAi

**Figure 2:**
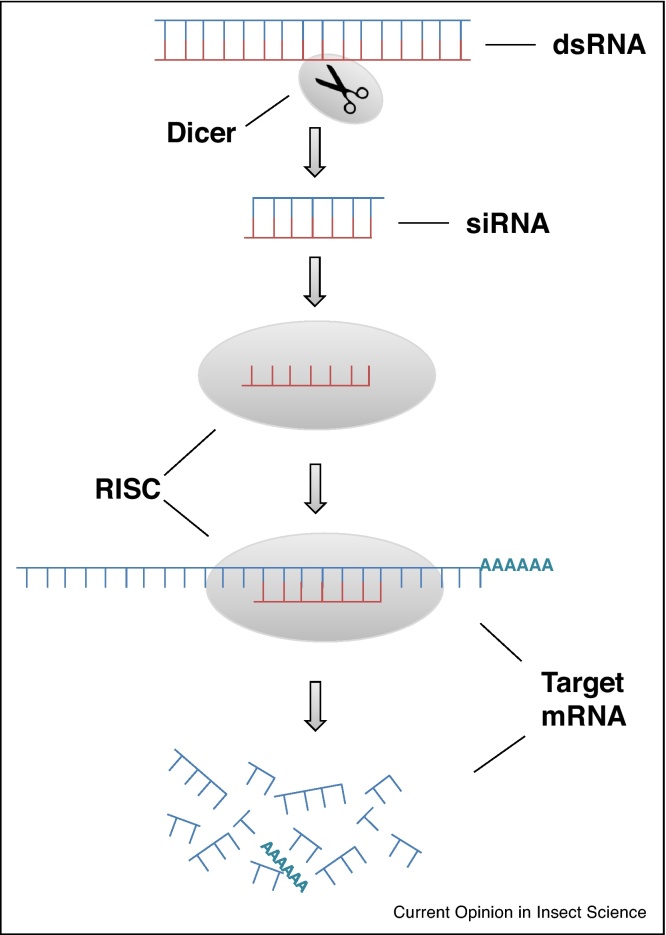
Basic mechanisms of RNA interference (RNAi). Double-stranded RNA (dsRNA) is cleaved into fragments of around 21 nucleotides (the small interfering RNAs, or siRNAs) by the enzyme Dicer. siRNAs antisense strands couple to the RNA-induced silencing complex (RISC) and convey it to target mRNA, blocking and degrading it.

RNAi interference (RNAi) is an evolutionary conserved gene silencing mechanism in which short interfering RNA (siRNA) molecules mediate sequence-specific degradation of messenger RNA (mRNA) before it is can be translated into polypeptide. It was first discovered in the free-living nematode *Caenorhabditis elegans*, when Fire *et al*. noted that introducing a double-stranded RNA (dsRNA) that was homologous in sequence to a specific gene resulted in the silencing of that gene [22]. The process starts when dsRNA is cleaved by a RNase III (Dicer) into 21–25 nt-long siRNA duplexes. These siRNAs are incorporated in the RNA-induced silencing complex (RISC), which discards the passenger strand, and binds to the target mRNA, cutting it and thereby hindering translation (Figure 2) [23, 24]. RNAi was rapidly adopted as a functional genomic tool as, in theory, the expression of any gene can be supressed provided the sequence of that gene is known. Another advantage of RNAi is its non-dependency on germ line transformation technologies. Instead, there are several different ways of delivering the dsRNA and siRNA. Most RNAi studies in non-transformable insect species have delivered dsRNA through either microinjection or feeding. However, other methods such as topical application [25], delivery *via* transgenic plants [26] and aerosolized siRNAs bound to nanoparticles have also been shown to be effective in some insects [27, 28]. Numerous factors can influence RNAi's performance and successful knock-down using RNAi has proven challenging in some organisms including several insect species [29, 30]. A recent report has linked the lower sensitivity of lepidopterans to RNAi to the up-regulation of an order-specific nuclease that is able to digest dsRNA before it is processed by Dicer into siRNA [31]. Despite the challenges, RNAi has been successfully employed in both nuisance and agronomic insect pests to study the mechanisms involved in insecticide resistance, particularly those mediated by detoxification enzymes.

To investigate the role of P450s in pyrethroid resistance in the bed bug *Cimex lectularius*, micro-injected dsRNA was used to knockdown *ClCPR*, the NADPH cytochrome P450 reductase required for the functioning of P450s. *ClCPR* knockdowns in deltamethrin-resistant populations caused a decrease in resistance to that insecticide, strongly suggesting that cytochrome P450s are involved in metabolising deltamethrin [32]. A similar dsRNA micro-injection approach was used in a series of elegant studies by Li *et al*. to decipher the involvement of the GPCR/Gαs/AC/cAMP-PKA signalling pathway in regulating resistance-related P450 gene expression in insecticide resistant populations of *Culex quinquefasciatus* [33, 34, 35]. In another mosquito species, *Aedes aegypti*, micro-injected dsRNA, targeting two Epsilon GST genes (*GSTe2* and *GSTe7*), was shown to cause a higher susceptibility to deltamethrin but not DDT in a resistant strain overexpressing these GST's [36].

The diamondback moth, *Plutella xylostella*, is a major pest of cruciferous vegetables, notorious for its ability to rapidly evolve resistance to insecticides. To investigate the molecular mechanism of pyrethroid resistance in *P. xylostella*, a droplet technique was employed to feed permethrin resistant fourth-instars larvae with dsRNA targeting the P450 CYP6BG1. The consequence of *CYP6BG1* knock-down in these larvae was an increase in susceptibility to permethrin [37]. RNAi has also been applied to investigate an insecticide resistance resurgence in a Florida population of the Asian citrus psyllid, *Diaphorina citri*, a vector of *Candidatus Liberibacter*, the causal agent of huanglongbing (one of the most destructive diseases of citrus). Concomitant knock-down of the expression of five *CYP4* genes previously implicated in resistance in this insect, by delivering dsRNA through topical micro-applications, increased the susceptibility of the insecticide-resistant populations to the neonicotinoid insecticide imidacloprid [25]. Similarly, knock-down of two P450s, CYP6AY1 and CYP6ER1 by microinjection of dsRNA, in the brown planthopper *N. lugens*, confirmed a functional role for these two enzymes in imidacloprid resistance [38]. For the cotton aphid, *Aphis gossypii*, carboxylesterase (*CarE*) expression and associated OP (omethoate) resistance was dramatically suppressed in resistant individuals following ingestion of dsRNA-*CarE* by oral sachet feeding (artificial diet and parafilm) [39]. In another elegant study, employing RNAi to knock down the expression of *CYP6BQ9* in *Tribolium castaneum* and the GAL4/UAS system to drive the expression of this gene in *D. melanogaster*, Zhu *et al*. provided compelling evidences demonstrating a major role for this P450 enzyme in deltamethrin resistance [40^••^].

## The CRISPR revolution

**Figure 3:**
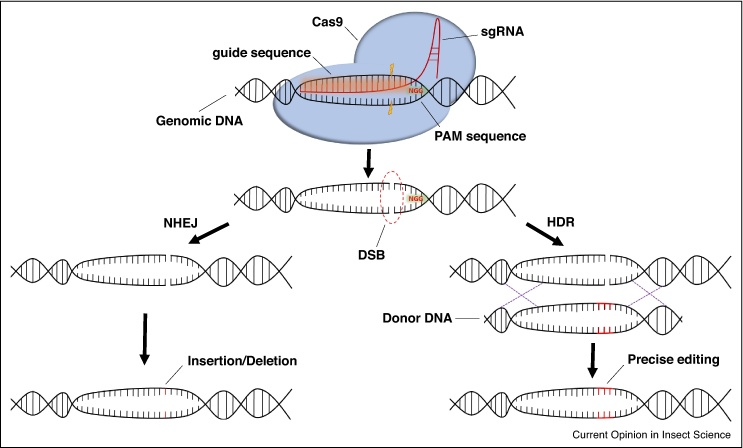
Schematic representation of the *Streptococcus pyogenes* Cas9 nuclease targeted to genomic DNA by a single-guide RNA (sgRNA) consisting of a 20-nt guide sequence and a scaffold (red). The guide sequence is directly upstream of the protospacer adjacent motif (PAM), NGG. Cas9 mediates a double-strand DNA break (DSB) 3 bp upstream of the PAM (orange highlights). The break is repaired either by non-homologous end joining (NHEJ), that can create random insertions or deletions at the target site, or homology-directed repair (HDR), that uses a donor DNA template during the process. Donor templates can be used to introduce precise modifications to the genomic DNA.

CRISPR/Cas (Clustered Regularly Interspaced Short Palindromic Repeats/CRISPR associated proteins) is an adaptive immune system found in bacteria and archaea that has been repurposed into a technology for editing the genome of other living organisms (for a perspective on the discovery and development of CRISPR/Cas as a genome editing tool see [41]). Cas9 is a DNA endonuclease associated with the CRISPR/Cas system found in *Streptococcus pyogenes*. This enzyme can be targeted to specific sequences of DNA by a short guideRNA molecule (gRNA), where it generates a double strand break (DSB) within that target site. Imprecise repair of DSBs can create null alleles. Alternatively, DSBs might be repaired by the homology-directed repair pathway, in which case, a donor template with homology to the damaged DNA can be manipulated to integrate specific alterations to that gene (Figure 3). CRISPR/Cas9 has proven a transformative technology, enabling directed, high precision genome modification of, and gene editing in, virtually any living organism [42]. Compared to other genome editing technologies such as ZFNs and TALEN, it is relatively simpler as it does not require repeatedly designing and expressing new nucleases. Instead, it only requires producing short target-specific gRNAs that associate with Cas9 to confer the desired site specificity, thus, making it an ideal laboratory tool. This system has already been successfully applied in several insect species (recently reviewed in [43, 44, 45, 46]). For the model insect *D. melanogaster*, the techniques are already very advanced, making it possible to precisely manipulate the genome in a way that leads to changes in gene expression and to the production of altered proteins [47]. The utility of this ground-breaking technology to investigate insecticide resistance mechanisms, in a defined genetic setting, is unparalleled and presents new and exciting opportunities to dissect the molecular basis of resistance (which may often be complex) into its component parts.

CRISPR/Cas9 has recently been used to investigate the mechanism underpinning resistance to spinosad, an economically important bio-insecticide. Resistance to this insecticide has already evolved in multiple pest insects and is associated with alterations of its target, the Alpha6 subunit of the nicotinic acetylcholine receptor (nAChRs). Chemical mutagenesis experiments in *D. melanogaster* identified the mutation P146S in *DmAlpha6* that conferred high levels of resistance to spinosad [48^•^]. To confirm the involvement of this mutation in the resistance phenotype, Somers *et al.* generated a CRISPR/Cas9-induced P146S fly strain resistant to spinosad [48^•^]. Taking a similar approach, Zimmer *et al.* functionally validated a candidate mutation (G275E) previously associated with field resistance to spinosad in the western flower thrips, *Frankliniella occidentalis* [49, 50].

Diamide insecticides, which are potent activators of insect Ryanodine Receptors (RyRs), are widely used to control lepidopteran pests. Resistance to these insecticides has been associated with mutations in the *RyR* gene of *P. xylostella* and the tomato leafminer, *Tuta absoluta* [51, 52, 53, 54]. To assess the contribution of three candidate resistance mutations G4946E, I4790M and G4946V to the resistance phenotype, CRISPR/Cas9 was employed to introduce these mutations in the *RyR* of *D. melanogaster*. G4946E caused lethality in transgenic flies and could not be assessed, whereas G4946V flies were viable and presented high levels of resistance to flubendiamide and chlorantraniliprole, and moderate levels of resistance to cyantraniliprole. Whilst wild type *D. melanogaster* already carries I4790M, the reversion of this, by gene editing, to M4790I induced higher levels of susceptibility to flubendiamide but less to chlorantraniliprole and cyantraniliprole [55^•^]. Although functionally inactive in gene edited flies, the G4946E mutation, when introduced by CRISPR/Cas9 into the beet armyworm, *Spodoptera exigua*, also conferred high levels of resistance to diamides [56^•^].

Benzoylureas (BPUs), buprofezin, and etoxazole are insect growth regulators classified as having different modes of action. A mutation (I1042M) in the *chitin synthase 1* (*CHS1*) gene of BPU-resistant *P. xylostella* was found to occur at the same position as the I1017F mutation in the two-spotted spider mite *Tetranychus urticae* conferring etoxazole resistance [57]. Using a CRISPR/Cas9 approach in *D. melanogaster*, Douris *et al*. introduced both substitutions (I1056M/F) into the corresponding *D. melanogaster CHS* gene. Homozygous lines bearing either of these mutations were highly resistant to etoxazole, BPUs and buprofezin, providing compelling evidence that all three insecticides share the same molecular mode of action and directly interact with CHS [58^••^]. Equivalent mutations (I1043M and I1043L) found in *Culex pipiens* mosquitoes resistant to the BPU diflubenzuron were also introduced into the *D. melanogaster CHS* gene using CRISPR/Cas9 and shown to confer significant levels (Resistance Ratio >2900 fold and >20 fold respectively) of resistance to BPU [59]. CRISPR/Cas9 has also been used to examine the relationship between detoxifying enzymes and pyrethroid resistance in *Culex quinquefasciatus.* When the cytochrome P450 gene *CYP9M10* was targeted in a resistant strain, the knockout individuals carrying no functional *CYP9M10* copy exhibited an ∼110-fold reduction in permethrin resistance [60^•^].

Transgenic crops expressing insecticidal toxins derived from *Bacillus thuringiensis* (Bt) are often the mainstay for the control of lepidopteran pest in several broad acre crops. Resistance to Cry1-type toxins is mediated by mutations in the midgut-associated cadherin (CAD) like protein and/or the ATP dependent binding cassette transporter ABCC2, both of which have been implicated as receptors for Cry1 protein in Lepidoptera. The role of CAD as a Cry1 receptor has been validated using a reverse genetic (CRISPR/Cas9) approach, where disruption of the CAD gene in a susceptible strain of *Helicoverpa armigera* led to a highly resistant phenotype [61], whereas for ABCC2 its role as a Cry1 receptor was identified using the GAL4/UAS approach in *D. melanogaster* [62]. Very recently, Jin *et al*. demonstrated the successful targeted genomic deletions of both CAD and ABCC2 genes in *H. armigera* with a mix of two gRNAs targeting different loci [63]. High levels of resistance to the Bt toxin Cry2Ab has been genetically linked with loss of function mutations in another ABC transporter ABCA2. A CRISPR mediated knockout of *H. armigera* ABCA2 was recently shown to confer high levels of resistance to not only Cry2Ab but also Cry2Aa [64].

## Perspectives and future directions

For biochemists and molecular biologists working in the field of understanding the fundamental basis of insecticide resistance, there has for far too long been a reliance on making correlative links between mutations or gene expression alterations and the resistance phenotype. However, advances in functional genomic technologies have made it possible for scientists to start testing those hypothetical correlations derived from genomic and transcriptomic studies. We are now able to functionally validate the role played by specific detoxification enzymes in the resistance phenotype of insects by employing heterologous expression (GAL4/UAS) and/or gene silencing (RNAi) systems. Furthermore, genome editing technologies such as CRISPR/Cas9 can be used to introduce any mutations that are implicated in insecticide resistance into living insects. Moreover, it is now possible to start combining various resistance mechanisms into controlled genetic backgrounds to assess their interactions and associated fitness costs.

*D. melanogaster* will keep pushing the frontiers in deciphering the molecular mechanisms involved in insecticide resistance, as the genetic toolkits developed for this model organism are still far more advanced than in any other insect. However, the development of germ-line transformation in non-model insects will facilitate these studies to be carried out in the pest insects. CRISPR/Cas9 will certainly play a pivotal role in this field of research and, in combination with other functional genomic technologies, will help decipher the molecular mechanism underpinning insecticide resistance.

## Conflict of interest statement

Nothing declared.

## References and recommended reading

Papers of particular interest, published within the period of review, have been highlighted as:

• of special interest
•• of outstanding interest
